# Bilateral Jugular Vein and Sigmoid Sinus Thrombosis Related to an Inherited Coagulopathy: An Unusual Presentation

**DOI:** 10.1155/2014/873402

**Published:** 2014-08-21

**Authors:** Özge Altıntaş, Azize Esra Gürsoy, Gözde Baran, Elnur Mehdi, Talip Asil

**Affiliations:** ^1^Department of Neurology, Medical Faculty, Bezmi Alem Vakıf University, Adnan Menderes Boulevard, Fatih, 34093 Istanbul, Turkey; ^2^Department of Radiology, Medical Faculty, Bezmi Alem Vakıf University, Adnan Menderes Boulevard, Fatih, 34093 Istanbul, Turkey

## Abstract

Internal jugular vein thrombosis (IJVT) is a rare condition associated with malignancy, coagulopathy, and trauma. The optimal management of any IJVT must be individualized and depends on the condition of the patient. *Case Presentation.* We report the case of a 42-year-old woman with a history of a first trimester spontaneous abortion. Apart from a tension-type headache, she had no neurological symptoms. She reported an incidental diagnosis of right-sided IJVT when she was evaluated for hyperthyroidism ultrasonographically. On ultrasonography, we observed bilateral jugular vein thrombosis. The patient was started on oral warfarin. Seven months later, when she was adequately anticoagulated, she developed a second thrombosis. According to the etiological workup, she had a mutation in the homozygous methylene tetrahydrofolate reductase (*MTHFR*) gene and reduced protein C levels and activity. *Conclusion.* This report illustrates an unusual presentation of a rare condition. In this case, the etiology was associated with the coagulopathy, which occurred despite adequate anticoagulation.

## 1. Introduction

Venous thrombosis affects mainly the lower extremities [1, 2]. Among the rare sites of venous thrombosis, the cerebral circulation is one of the most life-threatening sites. The symptoms vary and depend on the venous structure involved. The most common symptoms and signs of venous thrombosis of the cerebral circulation are headache and papilloedema due to intracranial hypertension [1, 3]. The prognosis of cerebral vein thrombosis (CVT) is favorable in more than 80% of cases, while poor neurological outcomes are seen in 7~20% and recurrence in 2.2~3% of patients [1].

Internal jugular vein thrombosis (IJVT) is an uncommon, potentially fatal, condition. The most common cause of IJVT is iatrogenic trauma [1]. Other recognized causes include malignancy, pregnancy, hormonal contraceptive therapy, and coagulation disorders [1]. A literature search found that bilateral IJVT or CVT has been reported rarely [4–6].

## 2. Case Presentation

A 42-year-old woman was admitted to our stroke clinic with a right-sided IJVT that was diagnosed incidentally upon ultrasonographic evaluation for hyperthyroidism. She complained only of a tension-type headache, which felt like a tight band around her head, unaccompanied by nausea or vomiting, and did not respond to paracetamol. Her physical examination revealed livedo reticularis on her limbs. Her neurological examination was normal. She had a history of a spontaneous first-trimester abortion. She had no contributory family history or oral contraceptive use.

**Figure 1:**
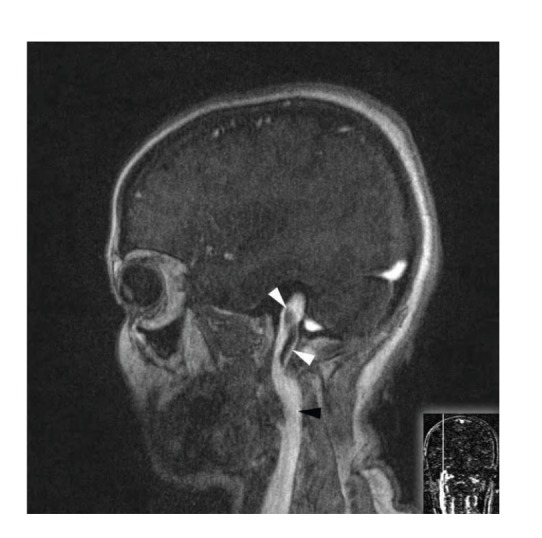
Right side: postcontrast MR T1W sagittal images clearly show a low-signal-intensity clot (white arrowheads) at the end of the left and right sigmoid sinuses extending along the jugular vein (black arrowhead).

**Figure 2:**
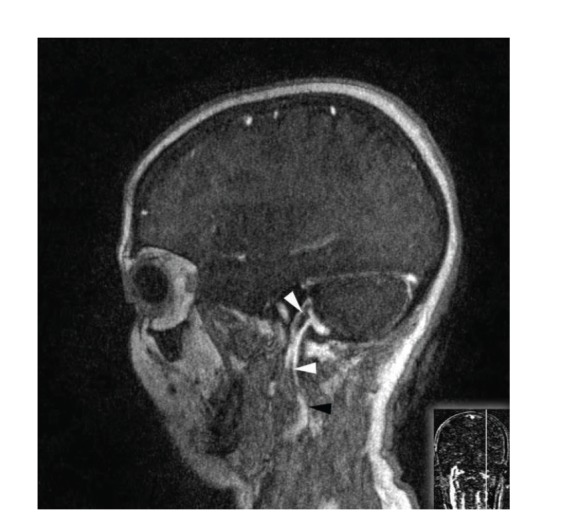
Left side: postcontrast MR T1W sagittal images clearly show a low-signal-intensity clot (white arrowheads) at the end of the left and right sigmoid sinuses extending along the jugular vein (black arrowhead).

We performed color-coded duplex Doppler ultrasonography to confirm the diagnosis of IJVT and observed bilateral jugular vein thrombosis. Magnetic resonance venography of the brain revealed bilateral internal jugular vein and sigmoid sinus thrombosis and a recanalized left transverse sinus thrombosis. The sagittal sinus and right transverse sinus were patent (Figures 1 and 2).

An etiological workup revealed a homozygous* MTHFR* gene mutation. No mutations in the prothrombin gene (*FII*) or Factor V Leiden (*FVL*) were discovered. IgM and IgG anticardiolipin antibodies and anti-dsDNA antibodies were negative. The homocysteine, protein S, and antithrombin III levels were within normal ranges. Only the protein C activity (49, normal range > 60) and level (20, normal range > 20) were lower than normal. A full cancer-antigen screening panel was examined, and the liver metabolic and synthetic functions were investigated fully; we found no abnormalities.

A diagnosis of bilateral IJVT, related to the homozygous* MTHFR* mutation and protein C deficiency, was established.

She was treated with warfarin initially, together with additional injectable low-molecular-weight heparin until effective. The international normalized ratio (INR) was maintained at 2.0–2.5. Seven months later, she was readmitted with a 2-day history of pain and swelling of the lower limbs without hyperemia or local heat. Her INR on admission was 2.18. A duplex ultrasound of her limbs identified left deep vein thrombosis. She was hospitalized for administration of intravenous heparin therapy during the acute stage, followed by oral anticoagulants. Her INR was adjusted to 2.5–3.0. The pain and swelling resolved over the course of a week, and her symptoms did not recur. She was discharged on oral anticoagulation.

## 3. Discussion

The frequency of bilateral IJVT is not known, but it has been reported to follow hemodialysis catheter placement, metastasis [5], trauma [6], and intravenous immunoglobulin infusion [4]. Our patient had IJVT related to an inherited coagulopathy, but no trigger factors were identified.

Thrombophilic abnormalities, either inherited (antithrombin, protein C, or protein S deficiency, with mutations in the* FVL*,* FII*, or* MTHFR* genes) or acquired (antiphospholipid antibodies) should be investigated in patients with CVT, as well as those with hyperhomocysteinemia [1].

We found that a homozygous* MTHFR* mutation and reduced protein C activity and levels were etiological risk factors. The reported incidence of* MTHFR* mutations in the general population ranges from 3% to over 40%. However,* MTHFR* mutations have only a very weak, if any, correlation with venous thrombosis [7].

Early anticoagulant treatment is crucial to limit thrombus extension. The American Heart Association/American Stroke Association (AHA/ASA) 2011 guidelines recommend anticoagulation using an oral vitamin K antagonist (VKA) and with a target INR of 2.0–3.0 for 3–6 months in patients with provoked CVT and for 6–12 months in those with unprovoked CVT. If the patient has recurrent episodes or homozygous mutations as risk factors, the duration of treatment should be individualized [3].

During the acute stage, the use of thrombolytic techniques and mechanical therapy is supported only by case reports and small case series. If clinical deterioration occurs despite the use of anticoagulation, or if the patient develops a mass effect from a venous infarction or intracerebral hemorrhage that causes intracranial hypertension resistant to standard therapies, then use of these interventional techniques should be considered [3].

For high-risk patients with inherited coagulopathies, the INR should be maintained at a higher level, between 2.5 and 3.0, and consideration should be given to long-term warfarin therapy.

Life-long treatment with VKA presents a risk of various bleeding complications, a narrow therapeutic range, and food-drug and drug-drug interactions. The recent availability of new oral anticoagulants provides alternative options.

The randomized, double-blind RE-COVER trial reported that a fixed dose of the direct thrombin inhibitor dabigatran is as effective as warfarin, with a similar safety profile, for the treatment of acute venous thromboembolism. There are no data to support the use of dabigatran monotherapy for acute cerebral venous thromboembolism [8]. Recently, Hon et al. reported two cases of CVT treated with dabigatran with good clinical and radiological outcomes [9]. Trials should examine the efficacy of new long-term oral anticoagulants.

## 4. Conclusion

In the case presented, unlike reported cases of bilateral IJVT, venous thrombosis was related to an inherited coagulopathy with no apparent clinical trigger factors.
